# “It was the whole picture” a mixed methods study of successful components in an integrated wellness service in North East England

**DOI:** 10.1186/s12913-018-3007-z

**Published:** 2018-03-22

**Authors:** M. Cheetham, P. Van der Graaf, B. Khazaeli, E. Gibson, A. Wiseman, R. Rushmer

**Affiliations:** 1 Health and Social Care Institute, Constantine Building, Teesside University, Middlesbrough, TS1 3BA UK; 2 Fuse (UKCRC Centre for Translational Research in Public Health), Newcastle University, Newcastle-upon-Tyne, NE2 4AX UK; 3 Gateshead Council, Public Health Team, Gateshead, NE8 1NN UK

**Keywords:** Integrated wellness services, Health and wellbeing, Commissioning, Social determinants, Lifestyle change

## Abstract

**Background:**

A growing number of Local Authorities (LAs) have introduced integrated wellness services as part of efforts to deliver cost effective, preventive services that address the social determinants of health. This study examined which elements of an integrated wellness service in the north east of England were effective in improving health and wellbeing (HWB).

**Methods:**

The study used a mixed-methods approach. In-depth semi-structured interviews (IVs) were conducted with integrated wellness service users (*n* = 25) and focus groups (FGs) with group based service users (*n* = 14) and non-service users (*n* = 23) to gather the views of stakeholders. Findings are presented here alongside analysis of routine monitoring data. The different data were compared to examine what each data source revealed about the effectiveness of the service.

**Results:**

Findings suggest that integrated wellness services work by addressing the social determinants of health and respond to multiple complex health and social concerns rather than single issues. The paper identifies examples of ‘active ingredients’ at the heart of the programme, such as sustained relationships, peer support and confidence building, as well as the activities through which changes take place, such as sports and leisure opportunities which in turn encourage social interaction. Wider wellbeing outcomes, including reduced social isolation and increased self-efficacy are also reported.

Practical and motivational support helped build community capacity by encouraging community groups to access funding, helped navigate bureaucratic systems, and promoted understanding of marginalised communities. Fully integrated wellness services could support progression opportunities through volunteering and mentoring.

**Conclusions:**

An integrated wellness service that offers a holistic approach was valued by service users and allowed them to address complex issues simultaneously. Few of the reported health gains were captured in routine data. Quantitative and qualitative data each offered a partial view of how effectively services were working.

## Background

The concepts of wellness and wellbeing are contested but are assuming an increasing role in the development of public health policy [1, 2]. Integrated wellness approaches acknowledge the multiple aspects of wellbeing which contribute to quality of life [3] and the complex social and economic influences which shape health related behaviour and decision-making [4].

In recognition of increasing pressures on public health budgets, a growing number of Local Authorities (LAs) have introduced integrated wellness services as part of efforts to deliver cost effective, preventive services, using asset based community approaches [5–9] (For example, see https://www.kingsfund.org.uk/sites/files/kf/chris-mcbrien-elspeth-anwar-knowsley-poster-mar13.pdf).

Moving away from a “silo” approach to commissioning and delivery of individual lifestyle services, integrated wellness services adopt a holistic approach, and seek to address the wider determinants of health [10, 11]. The aim is to support people to live well, building their capability to be independent, resilient and able to maintain optimum levels of health and wellbeing (HWB) for themselves and others in local communities [12], to reduce demands on health and social care services [13] and tackle inequalities in health [14].

Evidence of impact is emerging through evaluations of UK wellness services and social prescribing projects, which recognise the range of social, economic and environmental factors affecting people’s health and seek to address people’s needs in a holistic way. Reported benefits include improvements in perceived mental wellbeing [15], reductions in GP attendance rates [16], improved ability to manage long term conditions, and reduced hospital episodes [17].

However, the potential of integrated wellness services to address clustering of multiple unhealthy behaviours remains uncertain [18]. Evidence is inconclusive about the effectiveness of tackling multiple risk behaviours simultaneously or sequentially and in what combination [19, 20]. The challenges and limitations of health related behaviour change programmes were acknowledged by Kelly and Barker [21] who highlighted common errors in efforts to change behaviour, including the tendency to conceptualise behaviour as something that can be reduced down to things that individuals do and think as if they were isolated from other aspects of their lives. Other gaps in the evidence-base remain, including factors influencing user engagement, and the contribution such services make to addressing the social determinants of HWB [22]. Addressing inequalities in access is important as several studies have found a socioeconomic gradient in the prevalence of multiple risk factors associated with gender, income, class and educational achievement [18, 20].This study helps to address this gap by focusing on what helps people engage with integrated wellness services, with what effects.

Supporting the delivery of integrated, place based public health approaches remains one of Public Health England’s strategic priorities [23], but developing such services is challenging, as is devising methods to show if these services are working as planned. In this paper, we outline findings from a small scale, mixed methods evaluation of an integrated wellness service in the north east of England and discuss what was gained by delivering services in this way. We explored which components make a difference to service users and examined the effectiveness of current methods of capturing information on the operation of these services. We compared patterns of usage, and health gains captured in routinely collected monitoring data with the views of stakeholders. In the discussion, we comment on the adequacy of mechanisms available to assess these services, and their impact on service users' lives.

### The context

The north east of England faces particular public health challenges [24] with higher levels of deprivation, long term conditions and poorer healthy life expectancy than averages in England. In the study site, nearly 25% of the population live in one of the 10% most deprived areas of England [25]. Public health indicators related to mental health, smoking, alcohol-related harm, diet and nutrition, physical activity and levels of overweight are worse than England averages. 68.9% of Gateshead residents have excess weight, with highest levels in those aged 55–64 years, compared with England where 64.6% adults have excess weight. Obesity levels are higher than England averages (9.5% compared with 9.1%). Local survey data highlights wide variations of adult obesity across Gateshead where the proportion of obese adults in the 20% most deprived areas of England, is almost double that in the least deprived areas [26]. In 2015, 46.3% of adults reported levels of physical activity in accordance with Chief Medical Officer recommended guidelines, compared with 57% in England. Rates of antidepressant prescribing are high, compared both with the England average and with areas of similar deprivation [26].

#### Live well Gateshead - the Gateshead integrated wellness service

**Fig. 1:**
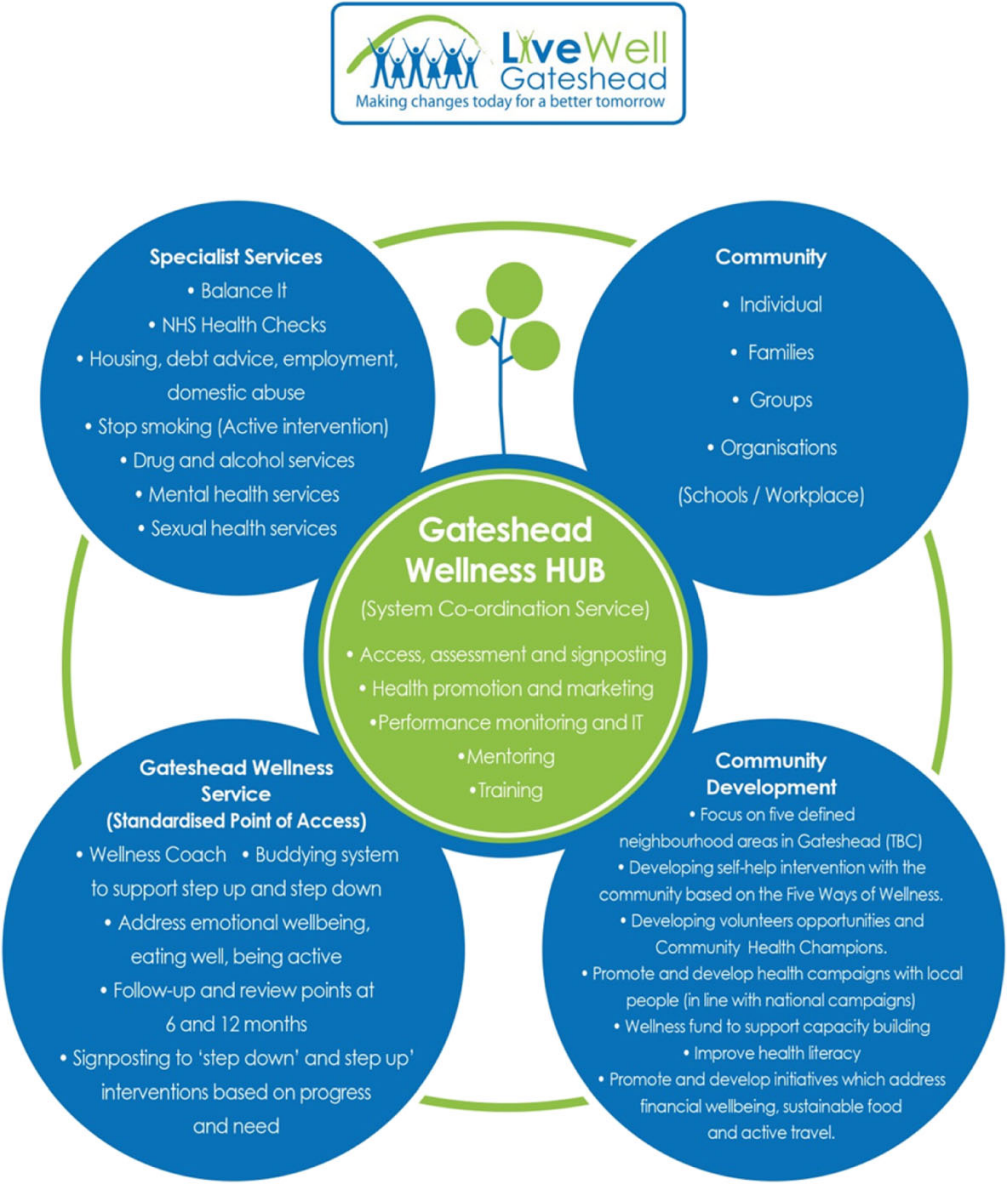
showing components of Live Well Gateshead reproduced with permission of Gateshead Council

Established in October 2014, the integrated wellness service, Live Well Gateshead (LWG), aimed to promote HWB, through a combination of tailor made, lifestyle interventions for individuals, groups, families and communities and improved service integration. Multiple services were offered at point of contact, by LA and NHS staff, through a clear triage and referral process. This included an initial assessment of need, up to twelve tailored 1:1 sessions with wellness coaches, group work, smoking cessation, weight management, dietary and healthy eating advice, physical activity pathways, mental health and emotional wellbeing interventions, alcohol brief interventions, signposting and accompanied referral to specialist agencies, such as welfare rights and housing advice, as shown in Fig. 1. Importantly, the model included a focus on the wider determinants of health. A combination of community development and community capacity building services were offered to support local community groups in developing their own solutions to issues they face. The service used a proportional universalism approach where the service was targeted at the 30% most deprived local communities.

## Methods

The study aimed to examine which elements of an integrated wellness service were effective in improving HWB in a Local Authority (LA) area in north east England. The theoretical framework used was realist evaluation to answer the overarching question of what works, for whom, under what circumstances [27]. It was important to understand which parts of the service were effective in engaging service users, what changes they made and how it was possible to assess these. Several data steams were used, including analysis of routinely collected statistics and performance monitoring data used by commissioners to understand patterns of service use and reported outcomes, alongside qualitative data collection.

### Routinely collected monitoring data

Approval was secured for the use of third party data from the data collection and reporting system (DCRS), which is a national system originally designed to capture data on the activities of health trainers [28], although questions have been raised about the accuracy and completeness of the data captured [29].

In the LA studied, data was routinely collected on specific measures to monitor the delivery of key performance indicators (KPIs) as outlined in the service specification established in the commissioning process. These formed part of the contract between the LA commissioners and include targets for the providers, which included other LA departments and NHS Trusts. In effect, this meant that the LA was both commissioner and provider of services. Many of these measures monitored adherence to the contract. Some were process measures (e.g. number of people accessing the service, for what, length of contact, etc.) and others measured health gains (e.g. weight loss or smoking cessation at 3, 6 and 12 months). Quantitative measures also included the shortened Warwick Edinburgh Mental Wellbeing Scale (SWEMWBS) which is a population-based, validated measure of mental wellbeing among people aged 16–74 in the UK [30]. This was designed to be completed at initial contact and at 3, 6 and 12 months, but in reality it was not administered to every service user and follow-up measures were often not reported.

Information on the activity of the 1:1 service interventions was entered onto DCRS, not that of the community development or community capacity building work, so an evaluation of these activities was only possible through other means, including the qualitative data.

### Qualitative data collection

To understand how the service was working ‘on-the-ground’ from the perspective of different stakeholders, primary qualitative data was collected to explore the experiences of service users, providers and commissioners. The aspiration was to understand stakeholders views, and to understand the factors influencing service user engagement and the ‘active ingredients’ that prompted change.

In-depth semi-structured interviews (IVs) with services users (*n* = 25), and three focus groups with non service-users (*n* = 23), were carried out. Participant information sheets and consent forms were posted to all those contacting the wellness service and working with the capacity building team between September – December 2015 inviting them to participate. Service user participants were aged 34–71 years and the majority (*n* = 20) were not in paid employment. Non-service users were recruited by emailing information about the study to people who had volunteered to be involved in the Council’s consultation processes. Three focus groups (FGs) with groups of service users (*n* = 14) took place, alongside observations of service delivery. Recruitment continued until data saturation was reached. In total, as shown in Table 1 below, 72 people took part in IVs and FGs lasting between 35 and 120 min. IVs and FGs took place in meeting rooms in the civic centre, local leisure centres and a local library. The qualitative data reported here is primarily from service users. Findings from the wider study, including IVs with staff delivering, managing or commissioning wellness services (*n* = 9) and observations of service delivery are reported elsewhere [9].

**Table 1:** showing data collected from September 2015 to May 2016

Method	Participants in total	Gender	
		Female	Male
Scoping and familiarisation meetings with staff in the Public Health Team, Community Capacity Building Team and Wellness Service, and with NHS Foundation Trust staff. Daily fieldwork notes were taken in these meetings.	17	Not recorded	
1:1 in-depth, semi-structured interviews with individuals who use LWG services	25	15	10
1:1 semi-structured interviews with LWG staff	9	6	3
1× focus group with LWG Healthy Lifestyle group members	4	4	0
2× focus groups with group members from 7 different groups in contact with CCBT members.	10	7	3
2× focus groups with people not in contact with LWG services	17	10	7
1× focus group with parents from a local primary school participating in a families programme	7	5	2
Total number of participants	72	47	25

Observations of training and group sessions included Emotional Health and Wellbeing Brief Intervention training delivered by NHS Trust staff, Healthy Lifestyle Programme sessions (*n* = 5) and Cooking on a Budget Course (*n* = 2) delivered by Wellness Coaches. Community Capacity Building Team (CCBT) staff were observed working with community groups and organisations (*n* = 3). These were chosen to represent a diverse selection of the group based activities underway and to help understand the LWG offer. All participants gave their informed consent to take part. All IVs and FGs were audio recorded, transcribed verbatim, thematically coded and analysed using framework analysis [31] to address the research questions. Participant feedback meetings, Advisory Group meetings and informal discussions with stakeholders helped to interpret and contextualise the findings, inform data analysis, recommendations and conclusions.

The findings reported in the following section include quotations from service users, indicated by SU, and non-service users views, indicated by NSU, designated male (M) or female (F) and from focus group participants indicated FG1, FG2 etc.

## Results

### Access and assessment

Analysis of routine monitoring data from DCRS (accessed 4th May 2016) shows that a total of 2121 individuals contacted the service between 1st October 2014 and 31st March 2016, and 1025 single holistic assessments were undertaken. Over half the service users (54%) heard about the service from their GP. 62% of wellness service users were over 46 years of age and 34% were recorded as living in the 20% most deprived wards.

The qualitative data showed that first contact was important. Some participants felt there should be a choice of telephone or face-to-face communication, and many noted the importance of a smooth, efficient, joined up process, which creates a positive first impression of the service:

*Initial contact should be inclusive and motivating and straightforward and everything happens when it should happen, and you can feel confident in it* (F, SU IV 11).

Discussions with non-service users (NSUs) revealed questions about the training and professional qualifications of LWG staff, as shown in the following quote:

*Who gives advice, what world are they from, the medical profession or what?* (MNSUFG4). NSUs felt concerns about confidentiality and embarrassment could put people off approaching the service:

*I think a lot of people might be slightly embarrassed having to ask for help. They’d rather suffer in silence I think. That’s the proud Geordies you see, don’t like asking for help* (F, NSUFG4).

Non-service users suggested promoting the service through trusted GP practices, voluntary sector organisations, Citizens Advice Bureau, Council News, and social media, as well as through schools, nurseries and existing community networks. Cost was an important issue to address, especially for families during the school holidays, given the need for prevention and early intervention: *You’ve got to get them young* (F, NSUFG5).

### Reasons for contact

Routine data showed that the overwhelming majority of LWG service users identified the primary reasons they contacted LWG were for diet (*n* = 282), exercise /physical activity (*n* = 249), weight loss (*n* = 102), weight management (*n* = 58), and smoking (*n* = 72), suggesting that LWG was operating primarily to address people’s concerns with diet and weight. However, during 1:1 IVs and focus group discussions, a number of complex psychosocial and economic issues emerged, including significant mental health concerns, anxiety and depression, stress, caring responsibilities, family worries, debt, unemployment, relationship problems, loneliness, social isolation, grief, loss and bereavement.

43% of wellness service users were recorded as having a disability on DCRS, which is relatively high compared to the 22% of the local population who reported a long term illness or disability which limited their day to day activities in 2011 census data. Taken together the data suggests that service users problems are multiple, complex and intertwined and that the quantitative routinely captured data did not tell the full story. It also suggested that, at least in part, LWG was reaching the target populations it was designed to reach.

Many interviewees sought help from LWG because of difficulties adjusting to illness, diagnosis or surgery or coping with transition and loss of confidence following significant life changes, redundancy, accidents or injuries. Some participants described the pressures of managing long term conditions, including asthma, diabetes, epilepsy, and living with chronic pain, which frustrated efforts to be active. A few participants described housing problems, and one interviewee was homeless at the time of IV. This suggested a range of highly complex, long standing issues behind initial presenting health concerns, raising questions about the optimum length of the programme:

*I’ve got what they call chronic depression, so it’s not going to go away. It’s about helping us manage it. And sometimes 12 weeks is not enough* (M, SU IV3)*.*

### What difference do wellness services make for individuals and communities?

DCRS records showed, of those who developed personal health plans (*n* = 629) from October 2014 – December 2015, 65% achieved their plan, 14% partly achieved it and 9% did not achieve it at 12 weeks. Outcomes were unknown for 78 people (12%). Routine monitoring figures indicated modest overall, aggregated health gains among service users at three months. For example, data from October 2014 – December 2015 showed improvements in average Body Mass Index (BMI) scores from 34.2 to 33.8 (− 1.2%) for 451 people, and in mental health and emotional wellbeing scores for 115 people (+ 12.4%) using the SWEMWBS. However, this data was incomplete with large amounts of missing cases and did not tell us how and why the improvements were made. In the following section, we explore the factors reported to influence service users’ progress towards meeting their self-identified goals.

### Factors influencing progress towards meeting goals

Table 2 shows the factors that influenced progress at individual, community and organisational levels. These are explored further in the following sections.

**Table 2:** Reports of what facilitated progress at individual, community and organisational levels

Individual level	Community level	Organisational level
Facilitators Holistic focus on mental and physical health and emotional wellbeing Clear communications and marketing of wellness offer Free / minimal cost Smooth, efficient, confidential access and assessment process Skills, knowledge and non-judgemental approach of wellness coaches Behaviour change techniques, including personal plan, goal setting, diary keeping, motivation, self reporting and feedback Choice of gender of coach Coaches who take time, do not push, or pressurise but offer informed, personalised, tailor-made advice and guidance Non-stigmatising, discrete services in safe, appropriate accessible local venues, available evenings and weekends Service promotes independence, builds confidence, self-esteem, self-efficacy and sense of purpose Service provides opportunities to grow and develop as individuals, and to progress Volunteering, buddying and mentoring opportunities are offered Support is offered to change old habits and overcome setbacks	Facilitators Resourcefulness and encouragement; finding out about local services, support to access different activities Informal, social interaction with peers in groups, making friends, reducing social isolation Meeting up and doing activities with partners and others in community Building strong, inclusive local networks and social connections with people Promoting inclusive, mutual support and assistance through caring positive relationships including with those facing additional barriers Sharing experiences, learning together and from one another Making plans collectively and as a community Feeling part of something Taking control, promoting local activism Developing new skills, trying new things Giving and sharing e.g. food, cooking skills, recipes, experiences Increasing resilience Support to access long term funding opportunities Good inter-organisational links, including with LA, voluntary organisations and primary health care staff in GP practices	Facilitators Shared understanding and acceptance of theory and ownership of principles underpinning integrated wellness model Senior level endorsement, strong, positive leadership Solid, mature, trusting working relationships Working environment which is secure, satisfying and rewarding for staff Commitment to genuine partnership and respectful collaboration Joined up connections between different elements of service Provision of funding and resources for VCS Clear data management and reporting systems Performance monitoring data used to inform service planning and delivery Robust systems in place for staff and service user feedback, which informs service developments Joint accountability and shared responsibility for outcomes Clear support and supervision structures and training opportunities in place for staff Successes celebrated and lessons learnt and shared

### What helped individuals’ progress?

The majority of participants talked in extremely positive terms about their relationship with their wellness coach, who *‘went the extra mile’* (F, SU IV2) and were appreciated for their flexible, supportive, non-judgemental approach and listening skills. Coaches’ recognition of the links between mental, physical, social and emotional health, diet and weight was important:

*I think the thing I was impressed with is that she realised it wasn’t just about my diet and losing weight, it was the whole picture. It was around how I felt mentally. It’s about my depression as well* (F, SU IV15).

Commenting on a desire to address a range of ‘*self-destructive behaviours’* and change all his ‘*bad habits*’ together, another interviewee commented:

*It seemed to be a way of rolling all that in to one. There’s several other changes I’m making to my life, so the more they come under one umbrella, the more chance I’ve got of actually seeing them through* (M, SU IV19).

Coaches recognition that health and social issues were inter-related appeared to help service users to address them. Interventions worked when services users could engage in ways that related to how they lived their lives. Some participants with long term conditions described feeling nervous and anxious about exercising, embarrassed about needing help and fearful about approaching services. Wellness coaches facilitated access to social, leisure and exercise opportunities for individuals, *and* their partners, friends and family, demonstrating the interconnected nature of people’s health related decision-making.

Participants described the benefits of activities that increased social connectedness, for example, regular local walking groups where ‘*it’s just slow and it’s green and everybody just chats’* (M, SU IV 4). Low self-esteem and lack of confidence were identified as barriers by non-service users, who felt that men faced particular barriers to access for mental health concerns, because of the stigma related to it: *Men like to think they’re the stronger species* (M, NSUFG5). It was suggested that men could be targeted in workplaces, unions, through gardening, allotments or men’s sheds:

*A lot of older men, they’ll not sort of mix, but you get them in a garden situation and they’re talking about their garden* (M, SU FG2).

Some participants mentioned how the service had improved their confidence to move back in to education, training and employment, and helped them to stay in work. One participant reflected on changes she made at work after identifying the cause of her stress levels and reported improvements in her diet, reductions in alcohol use and increased activities with her partner:

*When you’re less stressed, you don’t go home and drink a glass of wine… it’s made me open my eyes in a way to what I’ve been doing. I mean it’s not easy to change your habits, but I think it’s been really positive* (F, SU IV 5).

Interventions worked on multiple interconnected levels, and often had several beneficial effects. The mental health benefits of physical activity and exercise were identified by many people, alongside reduced social isolation and feeling part of the community. Asked what would be lost if the service was not available, one participant commented:

*I wouldn’t be exercising 2-3 times a week. I wouldn’t have had the confidence to go. I think people would be struggling more on their own, and probably feeling isolated, overwhelmed. I would feel a bit lonelier, not part of the community. I feel part of the community doing this* (F, SU IV20).

Other participants described feeling motivated to *‘take control and make changes’* (F, SU IV15) suggesting integrated wellness services had a role in promoting self-efficacy and supporting self-management. Overall, the qualitative data indicated a combination of physical, social, emotional, nutritional and educational benefits for participants, their partners and families. The wellness service offered support for participants, opportunities to reflect on their lives, talk about personal issues, ‘*offload*’*,* a chance to share experiences with others facing similar challenges, activities to look forward to, a reason for getting out, reducing social isolation and loneliness, promoting social networks, mental health and confidence, creating positive peer relationships, which in turn enabled health related lifestyle changes. These benefits were less clear in the routine quantitative data collected.

### What helped communities?

The LWG Community Capacity Building Team (CCBT) operated at different, complementary levels to the 1:1 service, by developing local support networks, activities and facilitating social connections. The team were seen to provide fresh ideas, confidence, motivation and re-assurance to community groups and organisations:

*You’ve got a vision in your head but it’s quite hard to know what to do, where to go, and (CCBT staff member) helped us make things happen* (F, SU FG3).

Participants described ways in which the team initiate, develop, support and sustain groups, help to navigate bureaucratic systems and ‘*covers all aspects including health, finance, housing and employment’*, to connect and build links between groups, using local knowledge:

*It’s all about them doing their homework, knowing what’s going on in the local area and physically bringing people together, being prepared to make face to face contact* (M, SU FG2)

Activities were offered which promoted interaction between generations, people living with and without disabilities, and mental health concerns, challenging negative stereotypes. ‘*Making connections and working together’* (F, SU FG3) encouraged understanding among participants of different life experiences, and raised awareness of the issues facing those living in poverty, refugee and asylum seeker communities, homeless people and young parents. There were examples of diverse community groups coming together to address holiday hunger, offer arts and sporting activities. Overall, participants valued the practical and emotional support they received from the CCBT, contributing to a sense of community cohesion and belonging:

*We’ve lost our community spirit and we need to bring it back. Live Well Gateshead is helping to bring it back* (F, SU FG2).

## Discussion

The data captured by routine data monitoring systems were shaped by requirements for tracking adherence to service specifications, as well as assessing health outcomes. Learning from the systems was limited by how the data is recorded and how complete it is. For example, the reasons people were recorded as engaging with LWG may appear to be different, but really be one (weight). Our findings replicated wider concerns with DCRS. Its utility has been questioned for underestimating the upstream factors affecting client health, such as housing and debt, and missing the wide range of impacts on service users [29]. Even if health measures were recorded consistently and accurately, the insights they generate may be limited. Traditional measures of HWB, like BMI and mental health, provided some insight in to the effects of integrated wellness services, but they were limited in their capacity to identify *why* improvements were made or what factors influenced service user engagement or explained how community services were working. In this study, qualitative data enhanced the capacity to understand the complexity of how, and why integrated wellness services worked as they did, and helped inform recommended changes to KPIs to improve LWG.

The qualitative data collection provided specific information on lifestyle changes made by LWG participants, and explored factors reported to facilitate progress towards intended goals. The paper identified examples of active ingredients, such as sustained relationships and confidence building, and activities through which these changes take place, such as group work, sports and leisure opportunities, which encourage social interaction, as well as wider wellbeing outcomes such as reduced social isolation.

The combined efforts of improving individual, social and community networks and providing opportunities for meaningful social participation, and the reported increased self-efficacy, enhanced self-esteem, and improved confidence and motivation, should not be under-valued as important HWB outcomes in their own right. These mechanisms were reported as essential ingredients that helped to improve perceived physical and mental health issues, and encouraged people to stay in work or move into education or employment. Relationships with wellness staff and community development workers reduced isolation, particularly at points of transition in life (e.g. retirement). These outcomes were recognised as health enhancing [14] and have been reported as cost effective in UK social prescribing interventions for specific populations [16, 17]. The difficulties of measuring social impact, not easily captured in traditional KPIs, have been recognised in related fields such as housing, where efforts have been made to overcome them (for example www.hact.org.uk/social-value-publications). These developments are in part motivated by legislative requirements on those commissioning services who have a duty to improve the economic, social and environmental wellbeing of an area (http://www.gov.uk/government/publications/social-value-act-information-and-resources).

While the majority of participants in this study initially approached the wellbeing service with concerns about diet and exercise, multiple complex issues emerged during in-depth IVs, that the service helped users to address. This highlighted the importance of a multi-skilled workforce, with appropriate support, training and referral pathways in place. It suggested that integrated wellness services which focused on the social determinants of health and facilitated access to multiple sources of support through different pathways was a sensible approach for commissioners to take.

Of particular importance was the message that change takes time. Wellness services which facilitated contact with people facing similar challenges, provided opportunities to learn from others through peer support. Movement through different elements of the wellness service was welcomed by participants, offering progression opportunities, and the chance to ‘give something back’, through volunteering or mentoring roles again building self-efficacy. Whether a service that was based on a 12-week programme could provide sufficient support for all service users to reach their goals was debateable, suggesting some flexibility may be helpful in meeting individual needs.

Analysis of routine monitoring data suggested some success in targeting and reaching communities facing greatest health inequalities, including older people with disabilities and people living in disadvantaged communities. The findings may have reflected higher levels of need or greater numbers of residents with disabilities in deprived areas, or reflected targeted efforts to recruit people with disabilities. Previous reviews found specific groups, including people with disabilities and mental health needs, did not engage with commissioned obesity and exercise services and completion rates were low at 1% of population [32], so the gains reported here suggest that LWG was reaching previously excluded groups.

The quantitative data shows lower numbers of men (36%) access the 1:1 wellness services, reflecting national DCRS data which reported that 68% of health trainer clients were women [28]. Ideas suggested to further increase the engagement of men included offering access to allotments and gardening opportunities, targeting sports venues and workplaces. The service offered choice of gender of the staff people worked with, which was welcomed.

Our findings indicated that participants valued a combined focus on physical and mental health and opportunities to address the wider determinants of wellbeing, with a range of 1:1, group work and community based interventions. What mattered was the approach of skilled staff, with knowledge of the local patch, offering confidential, non-judgemental, tailor made advice and support, delivered at the right pace, and available at major transition points in people’s lives. These findings endorsed studies on the role of lay health advisors which emphasised the need for person-centred, social and emotional support, alongside practical activities like walking, which promoted new social networks [12, 33, 34]. This acknowledged the diversity and complexity of people’s lives and the importance of sharing common concerns [35]. It also echoed previous studies which demonstrated that people who had success in changing one behaviour were more likely to be successful at changing others [36].

Designing holistic services which addressed multiple health-related behaviours alongside wider determinants of health was complex and required a joined up, whole system approach. Findings from the evaluation suggested that wellness services could make an important contribution to improving outcomes, when the multiple interconnected factors influencing HWB were recognised at individual, community and organisational levels. 1:1 interventions combined with effective group work and community capacity building opportunities appeared to make a difference as part of an integrated wellbeing offer, contributing to a sense of community cohesion and belonging. These observations were only made possible because of the qualitative data collection used to supplement the routine measures captured in DCRS.

### Strengths and limitations

This is a small scale, qualitative study and the views of those who participated in the research may not represent the views of all LWG service users and stakeholders. There is no way of knowing whether those who participated are in any way typical of all LWG service users, or groups. No families volunteered to participate, and more women than men participated in the research, reflecting patterns of LWG service use. Efforts were made to ensure a wide range of views and perspectives were captured in the research through open recruitment strategies, but self selection and use of gatekeepers to recruit participants may introduce the risk of bias. Caution should be exercised in interpreting the results in light of this.

## Conclusions

This paper contributes to growing evidence on the benefits and challenges of delivering integrated wellness services, including factors which facilitate engagement, influence service user progress, and improve community cohesion. The routine data capture told one story of selected health outcomes, whilst on-the-ground the reality of service user engagement, experience and health gains were more nuanced. Our findings suggest that KPIs are useful for service monitoring, but are not suitable for measuring the success of the whole programme. The evaluation suggested that integrated approaches offer opportunities to improve HWB at individual and community level. If a whole system approach is adopted, integrated wellness services can act as a catalyst for change addressing the root causes of ill-health and focusing attention on the interconnections between individual, community and organisational factors influencing HWB.

## Abbreviations

BMI: Body Mass Index
CCBT: Community Capacity Building Team
DCRS: Data Collection and Recording System
FG: Focus Group
HWB: Health and wellbeing
IV: Interview
JSNA: Joint Strategic Needs Assessment
KPI: Key Performance Indicator
LA: Local Authority
LWG: Live Well Gateshead
NHS: National Health Service
NSU: Non service-user
SU: Service user
SWEMWBS: Shortened Warwick Edinburgh Mental Wellbeing Scale
UK: United Kingdom
